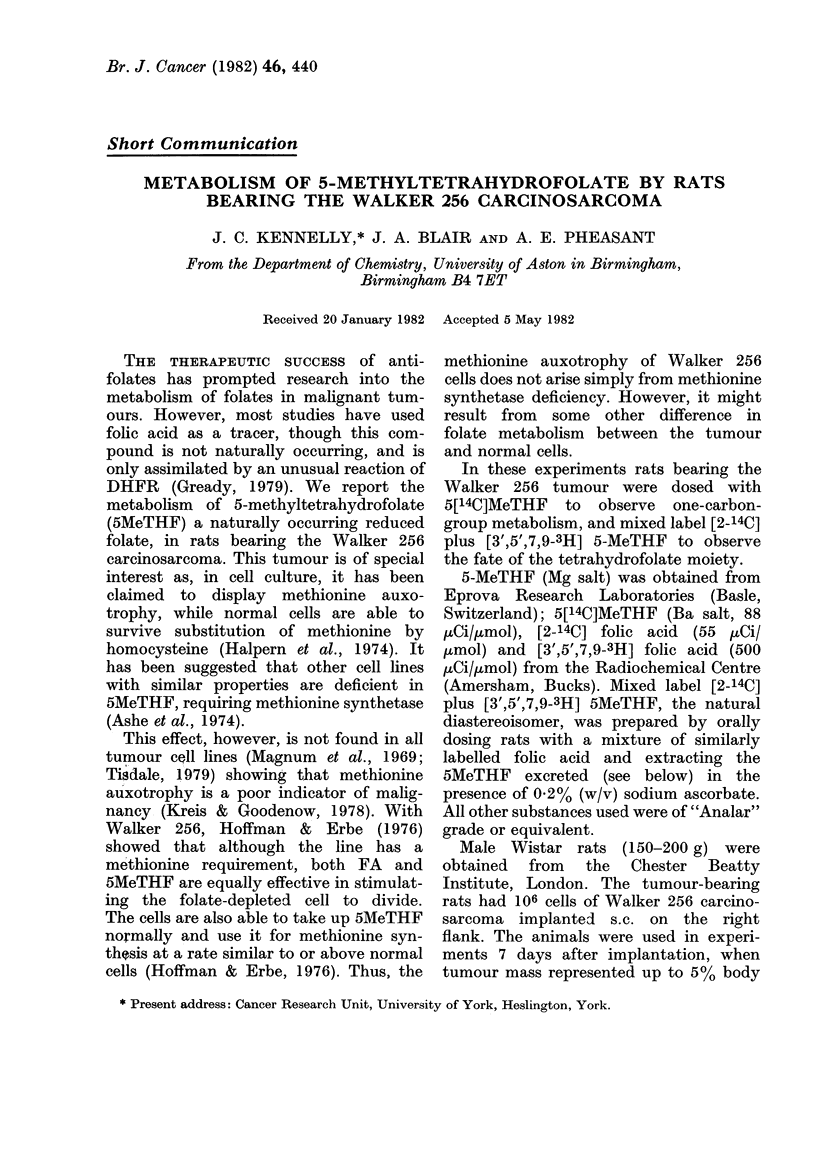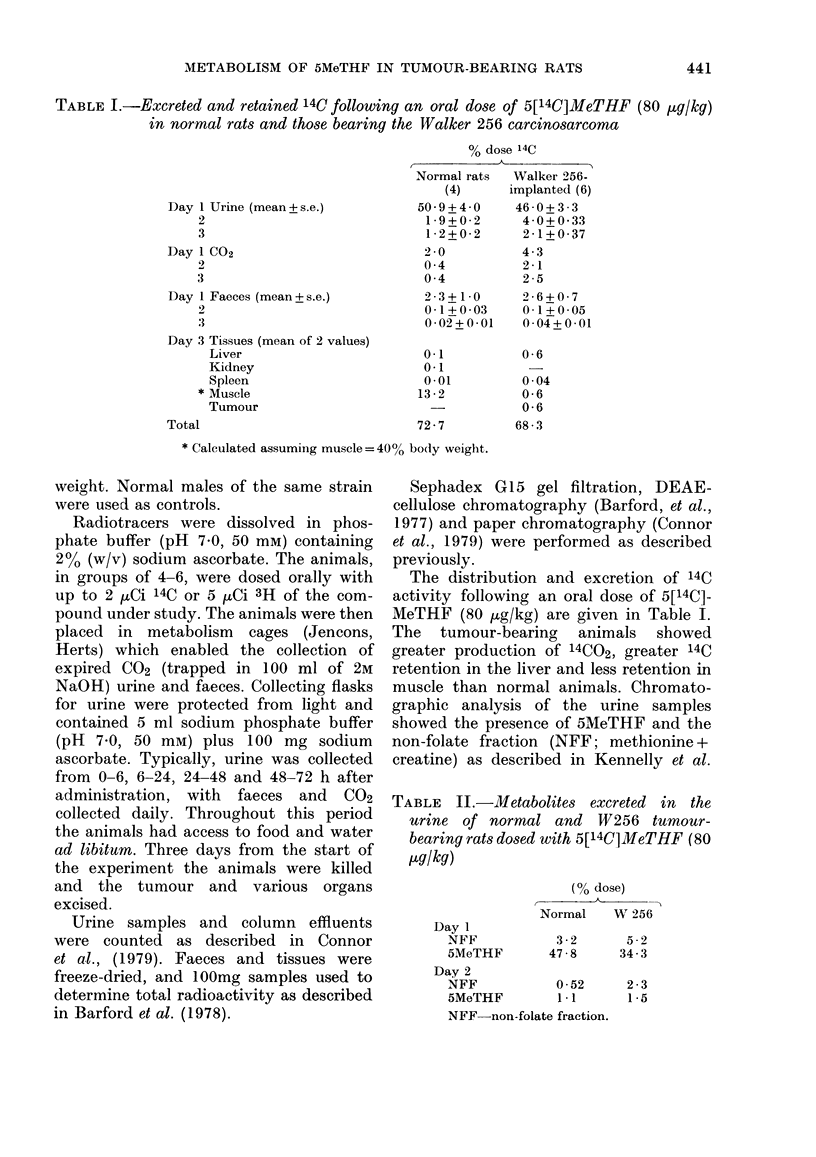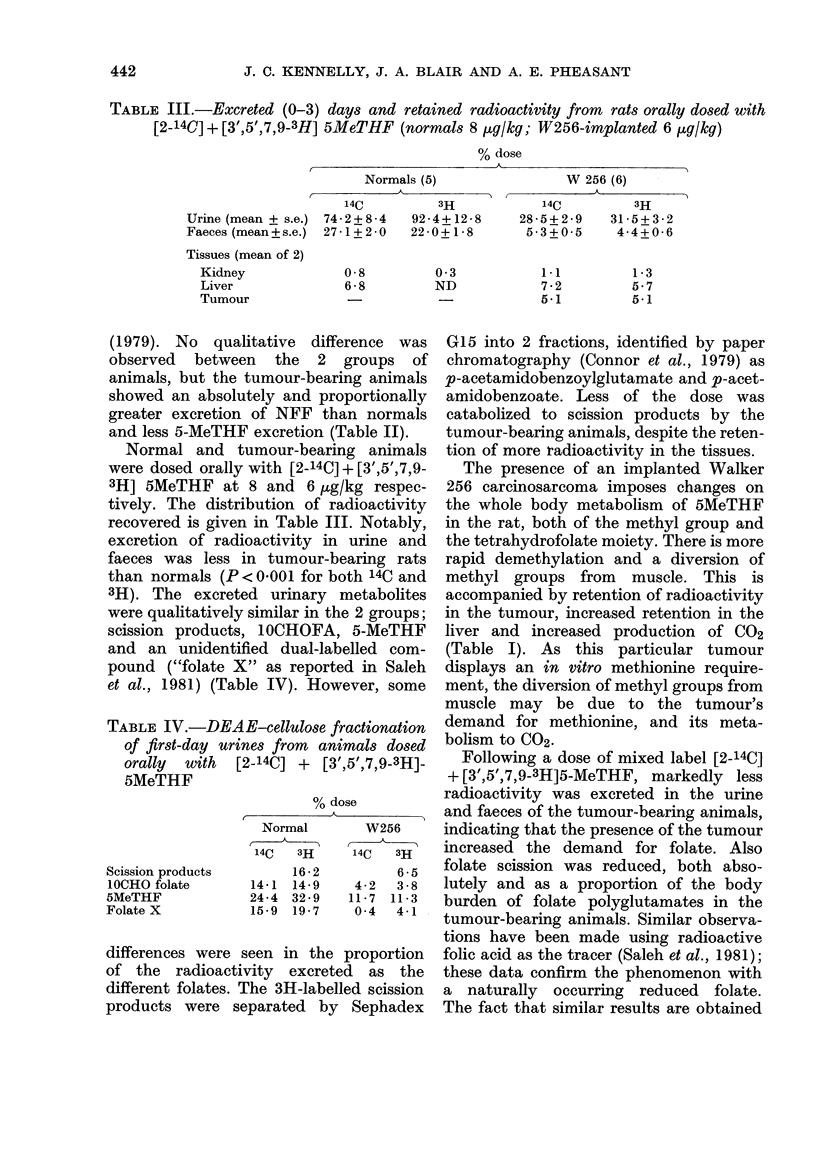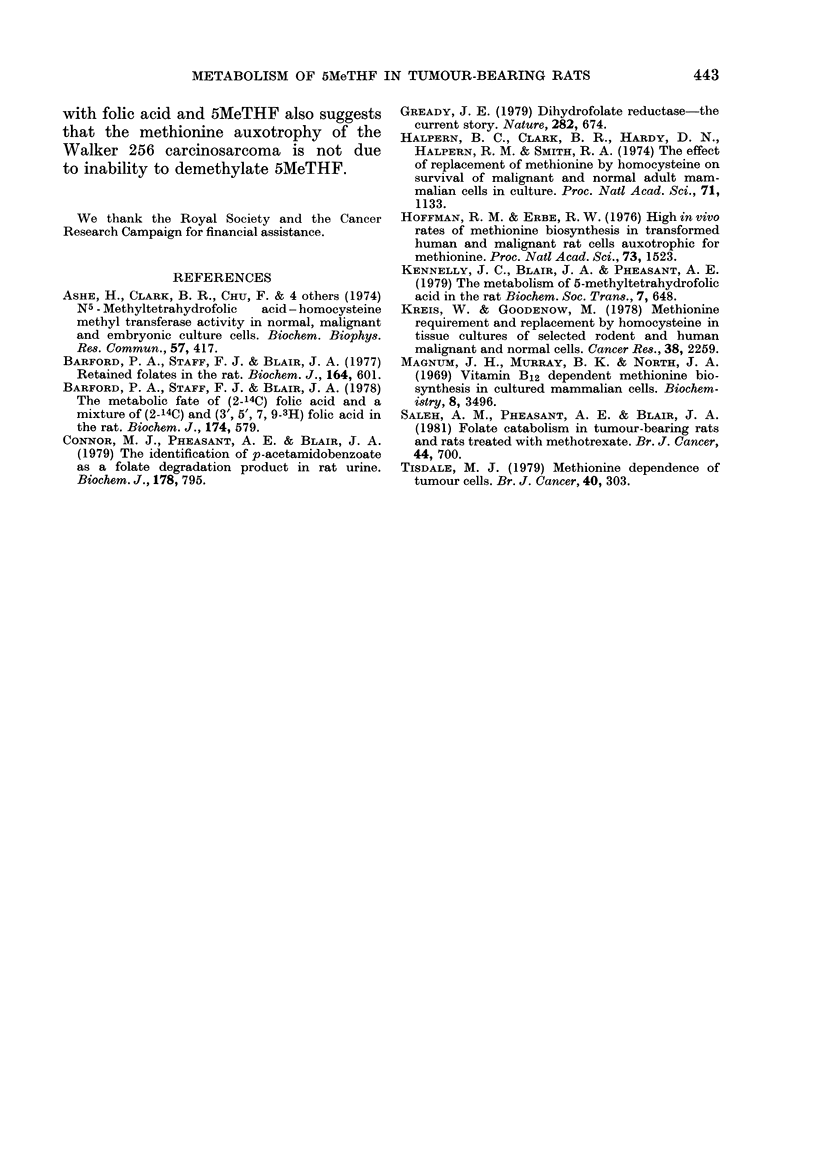# Metabolism of 5-methyltetrahydrofolate by rats bearing the Walker 256 carcinosarcoma.

**DOI:** 10.1038/bjc.1982.222

**Published:** 1982-09

**Authors:** J. C. Kennelly, J. A. Blair, A. E. Pheasant


*Br. J. Cancer* (1982) **46**, 440

*Short Communication*

### METABOLISM OF 5-METHYLTETRAHYDROFOLATE BY RATS BEARING THE WALKER 256 CARCINOSARCOMA

J. C. KENNELLY,* J. A. BLAIR AND A. E. PHEASANT

*From the Department of Chemistry, University of Aston in Birmingham, Birmingham B4 7ET*

Received 20 January 1982 Accepted 5 May 1982

THE THERAPEUTIC SUCCESS of antifolates has prompted research into the metabolism of folates in malignant tumours. However, most studies have used folic acid as a tracer, though this compound is not naturally occurring, and is only assimilated by an unusual reaction of DHFR (Gready, 1979). We report the metabolism of 5-methyltetrahydrofolate (5MeTHF) a naturally occurring reduced folate, in rats bearing the Walker 256 carcinosarcoma. This tumour is of special interest as, in cell culture, it has been claimed to display methionine auxotrophy, while normal cells are able to survive substitution of methionine by homocysteine (Halpern *et al.*, 1974). It has been suggested that other cell lines with similar properties are deficient in 5MeTHF, requiring methionine synthetase (Ashe *et al.*, 1974).

This effect, however, is not found in all tumour cell lines (Magnum *et al.*, 1969; Tisdale, 1979) showing that methionine auxotrophy is a poor indicator of malignancy (Kreis & Goodenow, 1978). With Walker 256, Hoffman & Erbe (1976) showed that although the line has a methionine requirement, both FA and 5MeTHF are equally effective in stimulating the folate-depleted cell to divide. The cells are also able to take up 5MeTHF normally and use it for methionine synthesis at a rate similar to or above normal cells (Hoffman & Erbe, 1976). Thus, the methionine auxotrophy of Walker 256 cells does not arise simply from methionine synthetase deficiency. However, it might result from some other difference in folate metabolism between the tumour and normal cells.

In these experiments rats bearing the Walker 256 tumour were dosed with 5[$^{14}$C]MeTHF to observe one-carbon-group metabolism, and mixed label [2-$^{14}$C] plus [3′,5′,7,9-$^{3}$H] 5-MeTHF to observe the fate of the tetrahydrofolate moiety.

5-MeTHF (Mg salt) was obtained from Eprova Research Laboratories (Basle, Switzerland); 5[$^{14}$C]MeTHF (Ba salt, 88 $\mu$Ci/$\mu$mol), [2-$^{14}$C] folic acid (55 $\mu$Ci/$\mu$mol) and [3′,5′,7,9-$^{3}$H] folic acid (500 $\mu$Ci/$\mu$mol) from the Radiochemical Centre (Amersham, Bucks). Mixed label [2-$^{14}$C] plus [3′,5′,7,9-$^{3}$H] 5MeTHF, the natural diastereoisomer, was prepared by orally dosing rats with a mixture of similarly labelled folic acid and extracting the 5MeTHF excreted (see below) in the presence of 0·2% (w/v) sodium ascorbate. All other substances used were of "Analar" grade or equivalent.

Male Wistar rats (150–200 g) were obtained from the Chester Beatty Institute, London. The tumour-bearing rats had $10^6$ cells of Walker 256 carcinosarcoma implanted s.c. on the right flank. The animals were used in experiments 7 days after implantation, when tumour mass represented up to 5% body

* Present address: Cancer Research Unit, University of York, Heslington, York.

TABLE I.—*Excreted and retained $^{14}C$ following an oral dose of 5[$^{14}C$]MeTHF (80 μg/kg) in normal rats and those bearing the Walker 256 carcinosarcoma*

| | % dose $^{14}C$ | |
|---|---|---|
| | Normal rats (4) | Walker 256-implanted (6) |
| Day 1 Urine (mean ± s.e.) | 50·9 ± 4·0 | 46·0 ± 3·3 |
| 2 | 1·9 ± 0·2 | 4·0 ± 0·33 |
| 3 | 1·2 ± 0·2 | 2·1 ± 0·37 |
| Day 1 $CO_2$ | 2·0 | 4·3 |
| 2 | 0·4 | 2·1 |
| 3 | 0·4 | 2·5 |
| Day 1 Faeces (mean ± s.e.) | 2·3 ± 1·0 | 2·6 ± 0·7 |
| 2 | 0·1 ± 0·03 | 0·1 ± 0·05 |
| 3 | 0·02 ± 0·01 | 0·04 ± 0·01 |
| Day 3 Tissues (mean of 2 values) | | |
| Liver | 0·1 | 0·6 |
| Kidney | 0·1 | — |
| Spleen | 0·01 | 0·04 |
| * Muscle | 13·2 | 0·6 |
| Tumour | — | 0·6 |
| Total | 72·7 | 68·3 |

* Calculated assuming muscle = 40% body weight.

weight. Normal males of the same strain were used as controls.

Radiotracers were dissolved in phosphate buffer (pH 7·0, 50 mM) containing 2% (w/v) sodium ascorbate. The animals, in groups of 4–6, were dosed orally with up to 2 μCi $^{14}C$ or 5 μCi $^{3}H$ of the compound under study. The animals were then placed in metabolism cages (Jencons, Herts) which enabled the collection of expired $CO_2$ (trapped in 100 ml of 2M NaOH) urine and faeces. Collecting flasks for urine were protected from light and contained 5 ml sodium phosphate buffer (pH 7·0, 50 mM) plus 100 mg sodium ascorbate. Typically, urine was collected from 0–6, 6–24, 24–48 and 48–72 h after administration, with faeces and $CO_2$ collected daily. Throughout this period the animals had access to food and water *ad libitum*. Three days from the start of the experiment the animals were killed and the tumour and various organs excised.

Urine samples and column effluents were counted as described in Connor *et al.*, (1979). Faeces and tissues were freeze-dried, and 100mg samples used to determine total radioactivity as described in Barford *et al.* (1978).

Sephadex G15 gel filtration, DEAE-cellulose chromatography (Barford, *et al.*, 1977) and paper chromatography (Connor *et al.*, 1979) were performed as described previously.

The distribution and excretion of $^{14}C$ activity following an oral dose of 5[$^{14}C$]-MeTHF (80 μg/kg) are given in Table I. The tumour-bearing animals showed greater production of $^{14}CO_2$, greater $^{14}C$ retention in the liver and less retention in muscle than normal animals. Chromatographic analysis of the urine samples showed the presence of 5MeTHF and the non-folate fraction (NFF; methionine + creatine) as described in Kennelly *et al.*

TABLE II.—*Metabolites excreted in the urine of normal and W256 tumour-bearing rats dosed with 5[$^{14}C$]MeTHF (80 μg/kg)*

| | (% dose) | |
|---|---|---|
| | Normal | W 256 |
| Day 1 | | |
| NFF | 3·2 | 5·2 |
| 5MeTHF | 47·8 | 34·3 |
| Day 2 | | |
| NFF | 0·52 | 2·3 |
| 5MeTHF | 1·1 | 1·5 |

NFF—non-folate fraction.

TABLE III.—*Excreted* (0–3) *days and retained radioactivity from rats orally dosed with* [2-$^{14}$*C*] + [3′,5′,7,9-$^{3}$*H*] *5MeTHF* (*normals* 8 *μg/kg; W256-implanted* 6 *μg/kg*)

| | % dose | | | |
|---|---|---|---|---|
| | Normals (5) | | W 256 (6) | |
| | $^{14}$C | $^{3}$H | $^{14}$C | $^{3}$H |
| Urine (mean ± s.e.) | 74·2±8·4 | 92·4±12·8 | 28·5±2·9 | 31·5±3·2 |
| Faeces (mean±s.e.) | 27·1±2·0 | 22·0±1·8 | 5·3±0·5 | 4·4±0·6 |
| Tissues (mean of 2) | | | | |
| Kidney | 0·8 | 0·3 | 1·1 | 1·3 |
| Liver | 6·8 | ND | 7·2 | 5·7 |
| Tumour | — | — | 5·1 | 5·1 |

(1979). No qualitative difference was observed between the 2 groups of animals, but the tumour-bearing animals showed an absolutely and proportionally greater excretion of NFF than normals and less 5-MeTHF excretion (Table II).

Normal and tumour-bearing animals were dosed orally with [2-$^{14}$C] + [3′,5′,7,9-$^{3}$H] 5MeTHF at 8 and 6 μg/kg respectively. The distribution of radioactivity recovered is given in Table III. Notably, excretion of radioactivity in urine and faeces was less in tumour-bearing rats than normals ($P < 0{\cdot}001$ for both $^{14}$C and $^{3}$H). The excreted urinary metabolites were qualitatively similar in the 2 groups; scission products, 10CHOFA, 5-MeTHF and an unidentified dual-labelled compound ("folate X" as reported in Saleh *et al.*, 1981) (Table IV). However, some

TABLE IV.—*DEAE-cellulose fractionation of first-day urines from animals dosed orally with* [2-$^{14}$C] + [3′,5′,7,9-$^{3}$H]-5MeTHF

| | % dose | | | |
|---|---|---|---|---|
| | Normal | | W256 | |
| | $^{14}$C | $^{3}$H | $^{14}$C | $^{3}$H |
| Scission products | | 16·2 | | 6·5 |
| 10CHO folate | 14·1 | 14·9 | 4·2 | 3·8 |
| 5MeTHF | 24·4 | 32·9 | 11·7 | 11·3 |
| Folate X | 15·9 | 19·7 | 0·4 | 4·1 |

differences were seen in the proportion of the radioactivity excreted as the different folates. The 3H-labelled scission products were separated by Sephadex G15 into 2 fractions, identified by paper chromatography (Connor *et al.*, 1979) as *p*-acetamidobenzoylglutamate and *p*-acetamidobenzoate. Less of the dose was catabolized to scission products by the tumour-bearing animals, despite the retention of more radioactivity in the tissues.

The presence of an implanted Walker 256 carcinosarcoma imposes changes on the whole body metabolism of 5MeTHF in the rat, both of the methyl group and the tetrahydrofolate moiety. There is more rapid demethylation and a diversion of methyl groups from muscle. This is accompanied by retention of radioactivity in the tumour, increased retention in the liver and increased production of $CO_2$ (Table I). As this particular tumour displays an *in vitro* methionine requirement, the diversion of methyl groups from muscle may be due to the tumour's demand for methionine, and its metabolism to $CO_2$.

Following a dose of mixed label [2-$^{14}$C] + [3′,5′,7,9-$^{3}$H]5-MeTHF, markedly less radioactivity was excreted in the urine and faeces of the tumour-bearing animals, indicating that the presence of the tumour increased the demand for folate. Also folate scission was reduced, both absolutely and as a proportion of the body burden of folate polyglutamates in the tumour-bearing animals. Similar observations have been made using radioactive folic acid as the tracer (Saleh *et al.*, 1981); these data confirm the phenomenon with a naturally occurring reduced folate. The fact that similar results are obtained with folic acid and 5MeTHF also suggests that the methionine auxotrophy of the Walker 256 carcinosarcoma is not due to inability to demethylate 5MeTHF.

We thank the Royal Society and the Cancer Research Campaign for financial assistance.

### REFERENCES

ASHE, H., CLARK, B. R., CHU, F. & 4 others (1974) $N^5$-Methyltetrahydrofolic acid-homocysteine methyl transferase activity in normal, malignant and embryonic culture cells. *Biochem. Biophys. Res. Commun.*, **57**, 417.

BARFORD, P. A., STAFF, F. J. & BLAIR, J. A. (1977) Retained folates in the rat. *Biochem. J.*, **164**, 601.

BARFORD, P. A., STAFF, F. J. & BLAIR, J. A. (1978) The metabolic fate of (2-$^{14}$C) folic acid and a mixture of (2-$^{14}$C) and (3′, 5′, 7, 9-$^{3}$H) folic acid in the rat. *Biochem. J.*, **174**, 579.

CONNOR, M. J., PHEASANT, A. E. & BLAIR, J. A. (1979) The identification of *p*-acetamidobenzoate as a folate degradation product in rat urine. *Biochem. J.*, **178**, 795.

GREADY, J. E. (1979) Dihydrofolate reductase—the current story. *Nature*, **282**, 674.

HALPERN, B. C., CLARK, B. R., HARDY, D. N., HALPERN, R. M. & SMITH, R. A. (1974) The effect of replacement of methionine by homocysteine on survival of malignant and normal adult mammalian cells in culture. *Proc. Natl Acad. Sci.*, **71**, 1133.

HOFFMAN, R. M. & ERBE, R. W. (1976) High *in vivo* rates of methionine biosynthesis in transformed human and malignant rat cells auxotrophic for methionine. *Proc. Natl Acad. Sci.*, **73**, 1523.

KENNELLY, J. C., BLAIR, J. A. & PHEASANT, A. E. (1979) The metabolism of 5-methyltetrahydrofolic acid in the rat *Biochem. Soc. Trans.*, **7**, 648.

KREIS, W. & GOODENOW, M. (1978) Methionine requirement and replacement by homocysteine in tissue cultures of selected rodent and human malignant and normal cells. *Cancer Res.*, **38**, 2259.

MAGNUM, J. H., MURRAY, B. K. & NORTH, J. A. (1969) Vitamin $B_{12}$ dependent methionine biosynthesis in cultured mammalian cells. *Biochemistry*, **8**, 3496.

SALEH, A. M., PHEASANT, A. E. & BLAIR, J. A. (1981) Folate catabolism in tumour-bearing rats and rats treated with methotrexate. *Br. J. Cancer*, **44**, 700.

TISDALE, M. J. (1979) Methionine dependence of tumour cells. *Br. J. Cancer*, **40**, 303.